# Ethanol extract of aloe vera ameliorates Artemether-Lumefantrine-induced reproductive toxicity in adult male Albino rats (Wistar Strain)

**DOI:** 10.5620/eaht.2023025

**Published:** 2023-11-17

**Authors:** Mustapha Olamide Oluwayemisi, Fatola Olanrewaju, Ola-Davies Olufunke, Ajayi Olusola Lawrence, Oyeyemi Matthew

**Affiliations:** 1 Department of Theriogenology, Faculty of Veterinary Medicine, University of Ibadan, Nigeria; 2 Department of Veterinary Anatomy, Faculty of Veterinary Medicine, University of Ibadan, Nigeria; 3 Department of Veterinary Physiology and Biochemistry, Faculty of Veterinary Medicine, University of Ibadan, Nigeria; 4 Department of Veterinary Pathology, College of Veterinary Medicine, Federal University of Agriculture, Abeokuta, Nigeria

**Keywords:** *Aloe vera*, Artemether-Lumefantrine, Testes, Immunohistochemistry, Spermiogram, Oxidative stress

## Abstract

Artemether-Lumefantrine (AL) is one of the alternative drugs used in treating malaria – an endemic scourge in Africa. AL has been reported to generate free radicals with long term use implicated in testicular pathologies. The antioxidative properties of *Aloe vera* (AV) has been well documented. This study investigated the ameliorative effect of ethanol extract of *Aloe Vera* on Artemether-Lumefantrine induced testicular toxicity. Thirty adult male albino rats were randomly divided into 5 groups (Control, AL-dosed, AV-dosed, AL+AV concurrently administered and AV-pretreated). Spermiogram, serum testosterone, testicular histopathology and inducible nitric oxide synthase (iNOS) immunohistochemistry were carried out. AL-dosed rats had poor spermiogram indices which were greatly improved in AV-dosed and AV-pretreated rats. These also corresponded with the testicular histopathology observations and were further buttressed by oxidative stress marker (iNOS) as AL-dosed rats had higher signal intensity compared to the control and AV-pretreated rats. Authors posit that concurrent administration of AV and AL protected testicular architecture while pretreating with AV prior AL administration improved the spermiogram. AL induces testicular pathology, thus should be used with care in male subjects. AV can confer a level of protection against these defects if used prior to administration of the drug.

## Introduction

Malaria is endemic in Africa, thus re-infection, recrudesce and recurrence is common [1,2]. Morbidity and mortality rates are rising in developing countries, largely due to the emergence of drug-resistant parasites rendering traditional antimalarial drugs such as chloroquine and sulfadoxine-pyrimethamine ineffective [3]. This put together encourages the prolonged use of antimalarial drugs. Artemether-Lumefantrine (AL) was introduced as one of the alternative drugs for the treatment of malaria in Africa [4-6]. AL has been reported to generate free radicals due to the presence of endoperoxide bridge [7,8]. Long-term administration of AL leads to accumulation of free radicals and therefore causes pathology in seminiferous epithelia and Leydig cells [9]; suppresses spermatogenesis [10], ultimately compromising sperm quality and function.

Evidence now suggests that reactive oxygen species (ROS)-mediated damage to sperm is a significant contributing pathology in 30–80% of cases of male infertility [11-14]. Tremellen [15] highlighted two key mechanisms of ROS by which it induces infertility in the male species: (a) ROS destroy the membrane of spermatozoa, thus reducing its motility and ability to attach to oocytes; (b) ROS compromise paternal contribution of genes to the embryo by directly destroying the DNA of spermatozoa. There are, however, ongoing efforts to search for novel natural antioxidants from fruits, vegetables, herbs and spices [16].

*Aloe vera*, AV (*Aloe barbadensis* Miller), a member of the family *Liliaceae*, is a short stemmed succulent, perennial herb with promising antioxidative potentials [17-22]. Anilakumar et al, [23] demonstrated that the organic extracts of AV leaf possess potent *in vivo* antioxidant capacity and this can prevent rat’s testicular tissue damage and promote spermatogenesis [24]. Similarly, the antioxidant potentials of AV have been demonstrated in the oxidative damage testicular tissues induced by other toxic chemicals and radiations, such as Bisphenol A, cisplatin and x-ray [25-27]. Mehrdad and Alireza [28] reported to have observed significant increase in the number of stem cells and primary spermatocytes in mice treated with AV extract. An important property of AV highlighted for this effect is probably its anti-apoptosis factor. The anti-apoptotic effect of AV can be ascribed to its antioxidant property as it reduces oxidation of fat and oxidative stress [29]. It also increases the natural defense of the body against oxidative stress by increasing the amount and effectiveness of innate antioxidant enzymes such as superoxide dismutase, glucose-6 -phosphate dehydrogenase and liver catalase [28].

Although much work has been done on the antioxidant potentials of AV [30-32], the ameliorative effect of AV on AL-induced testicular pathologies is yet to be ascertained. Therefore, this work seeks to investigate the effect of AL on the spermiogram, testosterone profile and testicular tissues of the albino rats (Wistar strain) and the putative ameliorative effect of AV on these testicular parameters.

## Materials and Methods

### Animals

Thirty (30) sexually matured male albino rats (Wistar strain) weighing between 170-180g were acquired from the Animal House of the Department of Zoology, Faculty of Science, University of Ibadan. They were housed in the animal house of the Department of Veterinary Anatomy, Faculty of Veterinary Medicine, University of Ibadan. The rats were allowed to acclimatize for two weeks, fed on pelletized rat feed and provided with water *ad libitum*.

### AL Preparation

The drug of choice for this study is Coartem® (20 mg Artemether and 120 mg Lumefantrine per tablet). Each tablet was dissolved in sterile water to make a solution of 1:6 mg/ml. The dosage employed in this study was 4:24 mg/kg body weight, administered orally with an oral gavage [33].

### AV Extraction

The plants were harvested in Ibadan and identified as Aloe barbadensis Miller with voucher number UIH-22461 at the Department of Botany, Faculty of Science, University of Ibadan, Ibadan. Extraction method adopted in this study was as described by Rajasekaran et al, [34]. The freshly harvested AV plant was washed with clean water to remove the adhering soil and dirt, cut transversely in pieces and then dried in a hot air oven at 60°C. The dried leaves were then grinded into powder with Marellex Excella® grinder. The powdered aloe sample with a weight of 282g was soaked in 1.5 liters 100% ethanol for 72hours on a shaker after which it was filtered and the filtrate was dried using Rotary evaporator RE52-2 Search Tech instrument at 40°C. Extract was then stored in a sterile air-tight container at 4°C until use. The extract was administered *per os* at the dosage of 100 mg/kg body weight as documented by Saritha and Anilakumar [35].

### Grouping and Treatment of Animals

Animals were randomly grouped into 5 (Groups 1, 2, 3, 4 and 5), with each group consisting of six sexually matured albino rats. Group 1 (Control) were given sterile water only *per os*. Group 2 (AL) received AL (4:24 mg/kg body weight *per os*) only for 6 days. Group 3 (AV): received AV (100 mg/kg body weight *per os*) only for 6 days. Group 4 (AV+AL concurrently) received AV (100 mg/kg body weight *per os*) following which AL (4:24 mg/kg body weight *per os*) was administered after 1 hour. This was done for 6 days. Group 5 [AV(pt)+AL] were pre-treated with AV (100 mg/kg body weight *per os*) for 7 days and subsequently, AL (4:24 mg/kg body weight *per os*) was then administered for the next 6 days. All animals were sacrificed at the end of each treatment according to the ethical norms of animal care and use research ethics of the Faculty of Veterinary Medicine, University of Ibadan, Nigeria.

### Serum Collection and Analysis

Venipuncture was done via the retro-orbital plexus at the medial canthus of the rat’s eye. Blood samples were collected into plain sample bottles using plain capillary tubes and left to stand at room temperature for about 1 hour. They were subsequently centrifuged using a bucket centrifuge at 1200 x g for about 10 minutes. The supernatants (sera) were collected into another plain sample bottle and stored at -20°C in a deep freezer for analysis. The sera collected were used for testosterone estimation using the Enzyme-Linked Immunosorbent Assay (ELISA).

### Organ Harvest

Prior to sacrifice, animals in each group were randomly subdivided into two sub-groups; each of the albino rats. Testes were harvested from the first sub-group for histopathology and immunohistochemistry while the testes (for testicular weight and volume) and epididymis (for semen analysis) were harvested from rats in the second sub-group.

#### Organ Harvest for Histopathology and Immunohistochemistry

Rats in the first sub-groups were anaesthetized with ketamine and transcardial perfusion was done. Concisely, with the rat placed on dorsal recumbency, a ventral midline incision was made craniocaudally on the chest region to open up the thoracic cavity so as to expose the heart. A scalp vein needle was used to introduce the saline/fixative into the left ventricle while a slit was made on the right atrium so as to allow the drainage of the blood out of circulation. Normal saline (0.9%) was first used until the return flow of the saline through the right atrium was clear and then 10% buffered formalin was used until the signs of perfusion were noticed, for example, paleness of the liver, fluid coming out of the natural orifices and rigor. After perfusion, the testes were harvested. The left testes were post-fixed in Bouin’s fluid for 72 hours while the right testes were post-fixed in 4% buffered formalin for 48 hours for histopathology and immunohistochemistry respectively.

#### Haematoxylin and Eosin Stain

Testicular tissues for histopathology were processed and stained with H&E in accordance with Wilson [36]. Tissues were dehydrated in ascending grades of ethanol, embedded in paraffin wax, and sectioned. Subsequently, de-waxed with xylene, and then rehydrated in descending grades of ethanol. Haematoxylin stain was added for 6 minutes. Slides were decolorized in acid ethanol then counter stained with eosin. They were subsequently dehydrated in ascending grades of ethanol, cleared in xylene, mounted and cover-slipped.

#### iNOS Immunohistochemistry

Testicular tissues were processed for immunohistochemistry based on the methods described by Farombi et al, [37]. Testicular sections were dipped in 4% phosphate buffer formalin. Antigen retrieval was done in 10 mM citrate buffer for 20 minutes, with subsequent peroxidase quenching in 3% H2O2/methanol. All the sections were blocked in 2% skimmed milk overnight and probed with the following antibodies: anti-iNOS antibody (rabbit polyclonal antibody, catalogue no. 611473; Transduction Laboratories, San Diego, California, USA; at a dilution of 1:200) for 16 hours at 4°C. Detection of bound antibody was done using appropriate HRP-conjugated secondary antibodies in VECTASTAIN kit (Vector Labs, Burlingame, USA) according to manufacturer’s protocol. The reaction product was enhanced with diaminobenzidine (DAB) for 6-10 minutes, with subsequent dehydration in ethanol, clearing in xylene and mounting on frosted glass slides. All images were captured using AmScope® Digital Camera Software (Version: x64, 3.7.3036). iNOS signaling intensities were quantified using the Image J software application (1.46r version).

#### Organ Harvest for Semen Analysis

Animals in the second sub-groups were euthanized by quick cervical dislocation, placed on dorsal recumbency and a ventral midline incision was made along its linea alba. The intra-abdominal testes (left and right) were excised and immediately weighed using LARK® LP502A electronic weighing scale. Testicular volume, epididymal sperm characterization and morphological studies were carried out as described by Oyeyemi et al. [38].

### Statistical Analysis

Data were presented as mean ± SEM and analysed with One-way Analysis of Variance (ANOVA) using GraphPad Prism 7.0. Tukey’s *post hoc* test was carried out to compare the statistical difference in means across all groups with statistical significance set at p ≤0.05.

## Results

### Testicular Parameters (Mean Testicular Weight and Volume)

**Figure 1.**
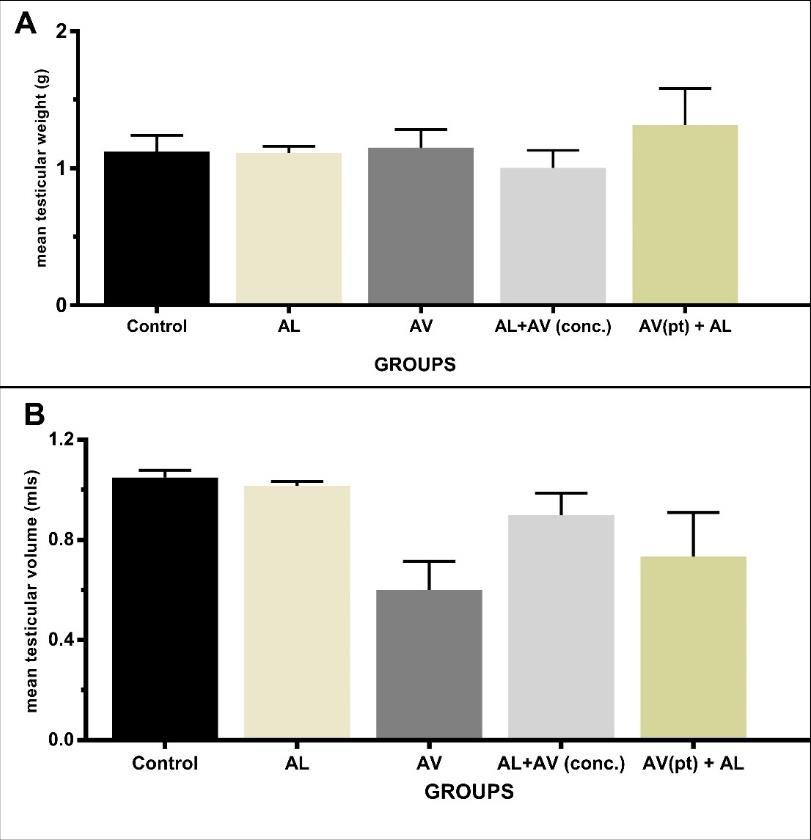
Comparison of (A): mean testicular weight and (B): mean testicular volume of albino rats across groups. Values are expressed as means ± SEM. No statistical significance in the mean values.

There was no statistical significant difference in the mean testicular weight across all experimental groups (Fig. 1a). Although animals dosed with AV only had the least testicular volume, this parameter was not statistically significant across all groups (Fig 1b).

### Semen Analysis

**Figure 2.**
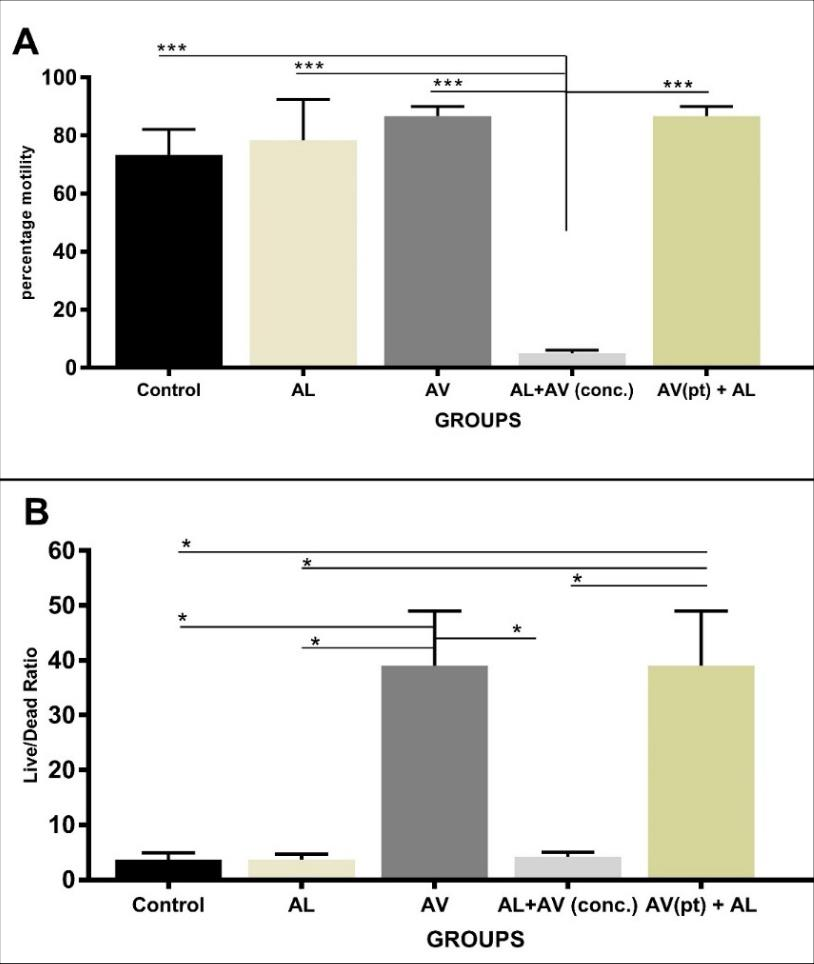
Comparison of (A): mean epididymal sperm motility and (B): mean sperm livability in albino rats across all groups. Statistical significance set at p≤.0.05. Values are expressed as means ± SEM. * indicates statistical significance at P≤ 0.05; *** indicates statistical significance at P≤ 0.001

Sperm motility in AV only and AV(pt) groups showed higher values and were statistically significant compared to groups administered concurrently AL and AV. The latter had the least score for sperm motility and was statistically significant when compared to other groups (Fig. 2a). Sperm cells live/dead ratio was significantly higher in rats dosed with AV alone and those pre-treated with AV as compared to other groups. There is, however, no statistical significant difference between AV alone and AV pre-treated groups (Fig. 2b).

**Figure 3.**
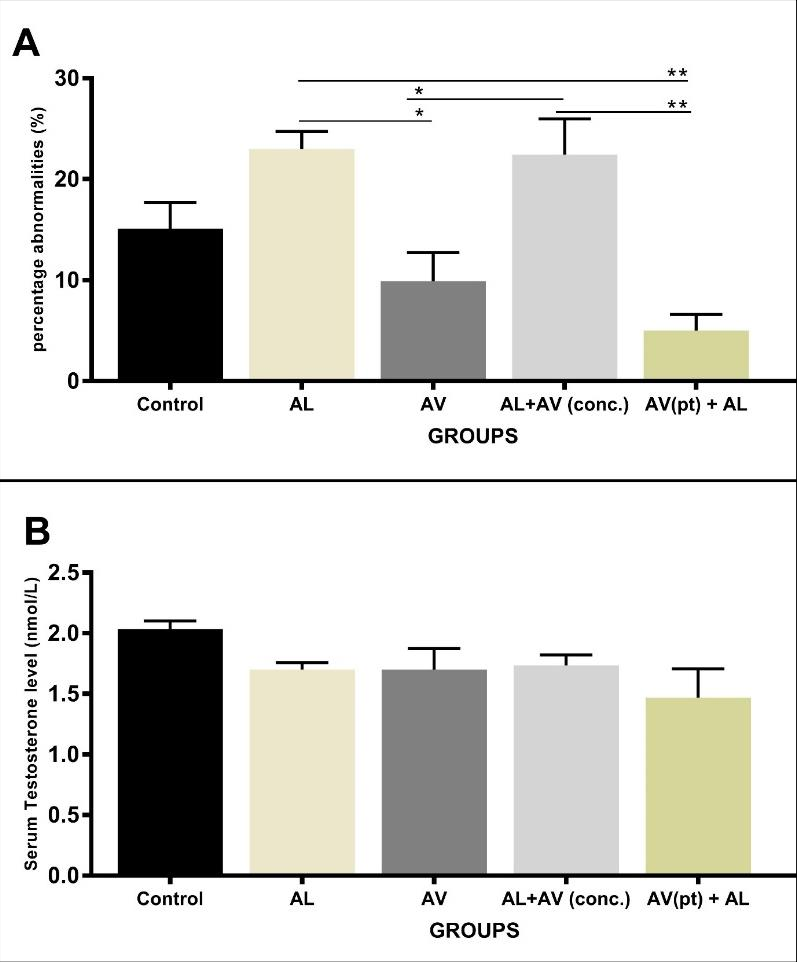
Comparison of (A): mean percentage abnormalities and (B): mean serum testosterone levels in albino rats across all groups. Statistical significance set at p≤.0.05. Values are expressed as means ± SEM.

Higher percentage abnormalities in sperm cells were seen in animals dosed with AL alone and in animals concurrently administered with AL and AV. These percentages were statistically significant when compared to controls, AV and AV (pt)+AV groups (Fig. 3a). The spectra of abnormalities seen ranged from bent tails, looped tails, detached tails, coiled tails and curved mid-pieces. Animals dosed with AV alone had lower percentage abnormalities compared to controls, although differences were not statistically significant at p<0.05. The least percentage abnormalities were recorded in groups pre-treated with AV prior to the administration of AL.

### Serum Testosterone

Mean serum testosterone levels (measured in nmol/L) was highest in control groups, while AL alone, AV alone and AL+AV(conc.) groups all had similar values. AV (pt)+AL groups had the least serum testosterone levels (Fig. 3b). However, there were no statistical significant differences in these values across all groups.

### Histopathology

**Figure 4.**
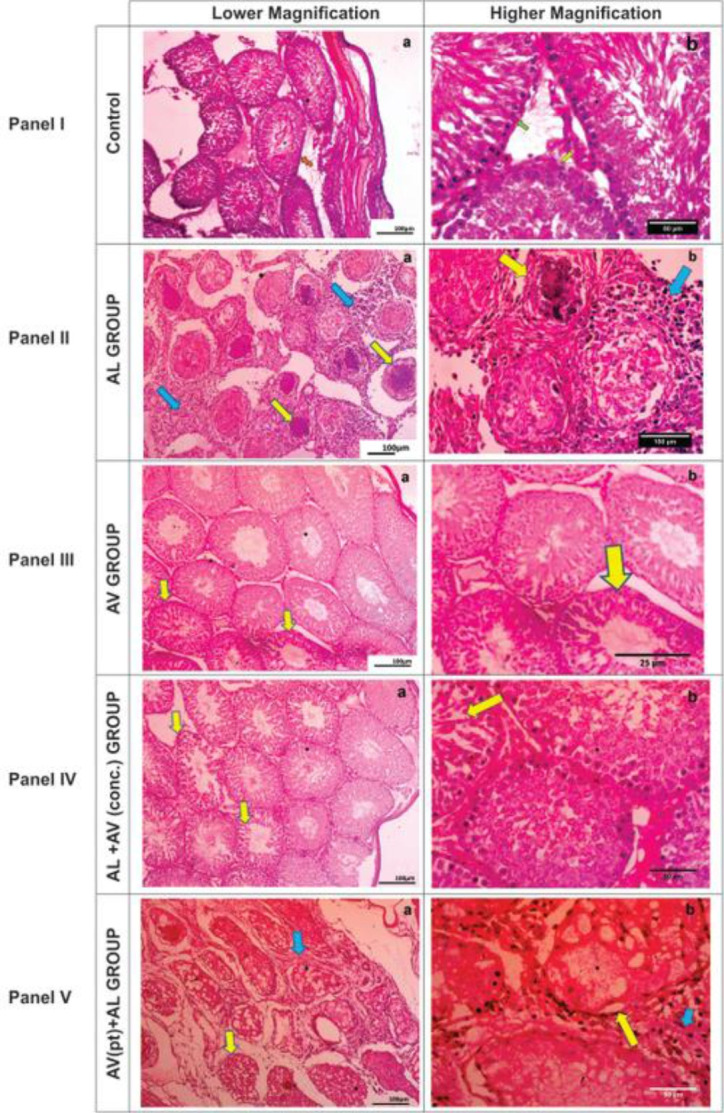
Photomicrograph of testicular pathology of albino rats across groups. a: Lower magnification; b: Higher magnification.

Testicular micrographs of control groups revealed normal cytoarchitecture of the seminiferous tubules with intact germinal epithelia and interstitial endocrine (Leydig) cells (Fig. 4 Panel I).

However, AL-dosed rats (AL) showed marked degeneration and necrosis of the germinal epithelial cells of the seminiferous tubules, mineralization of their luminal contents with no evidence of spermatogenesis (spermiostasis). The basement membranes of the seminiferous tubules were equally thickened, with moderate diffused granulomatous reactions in the interstitium (Fig. 4 Panel II). AV-dosed (AV) rats’ testicular histomorphology was similar to that of the control group with the evidence of spermatogenesis and intact interstitial cell populations. However, mild desquamation of the germinal epithelium was noticed (Fig. 4 Panel III).

There was an improvement in the histological picture of AL+AV(conc.) dosed rats with preservation of the seminiferous tubules and interstitium integrity in comparism to AL-dosed rats (Fig. 4 Panel IV). Rats pre-treated with AV prior to AL administration showed slight disruption in the histoarchitecture of the seminiferous tubules compared to ALdosed rats (Fig. 4 Panel V).

Panel I: Rat testis of control showing normal cytoarchitecture of the germinal epithelium, Sertoli cells (green arrow), intact basement membrane (orange arrow) and interstitial Leydig cells (green arrow). Panel II: Rat testis of AL alone (AL) showing marked degeneration, necrosis and mineralization of the germinal epithelium (yellow arrows) and moderate diffuse granulomatous reaction in the interstitium (blue arrows). Panel III: Rats dosed with AV alone (AV) showing apparently normal testicular cytoarchitecture with mild degeneration of the germinal epithelial cells (yellow arrows). Panel IV: Testicular morphology of rats dosed with AV+AL (conc.) with a near normal histology with very few degenerating germinal epithelia seen. Panel V: Rats pre-treated with AV prior to AL administration revealed reduced/milder degeneration of seminiferous tubules (yellow arrows) and granulomatous reaction in the interstitium (blue arrows) compared to AL group.

### iNOS Immunohistochemistry

**Figure 5.**
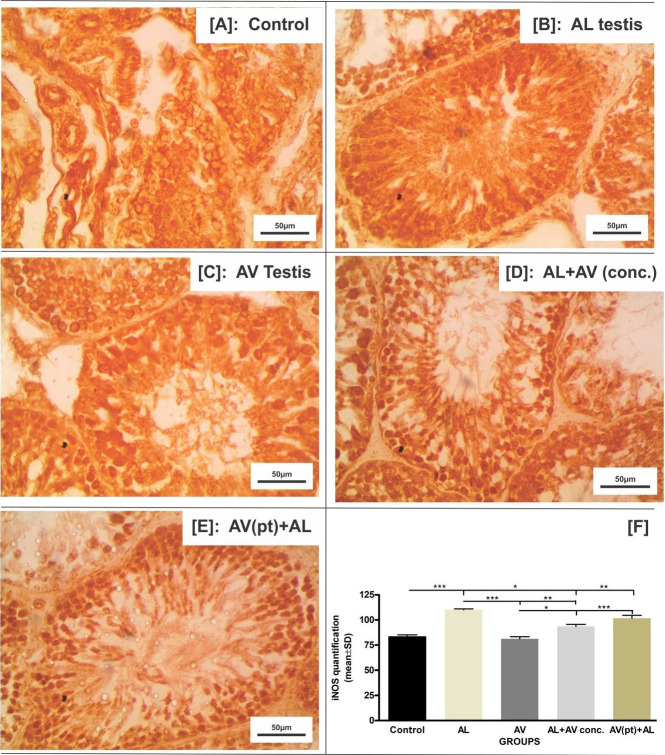
(A-E) Immunohistochemical signaling of inducible Nitric Oxide Synthase (iNOS) in the testis of Wistar rats across all groups; (F): Quantification of signal intensity using FIJI. Signal intensity was measured as mean ± SD. Statistical significance: * = p< 0.05; ** = p<0.01; *** = p<0.001.

AL treated group had the highest and most striking signal intensity values per unit area compared to control, AL+AV conc. and AL+AV pretreated groups. AV treated groups had the least signal intensity. Rats in group pre-treated with AV for 7 days prior to AL administration, showed signal intensity values very close to the control group (Fig. 5).

## Discussion

This work documents the ameliorative effects of ethanolic extract of Aloe vera on spemiogram and testicular tissues in adult male albino rats, following repro-toxicity caused by Artemether-Lumefantrine administration in adult male albino rats.

Results from this work showed no significant difference in the mean testicular volume and weight across all the groups. This agrees with the findings of Oyewopo et al. [39] in Sprague-Dawley rats. Motility of the sperm cells in animals treated with AV alone and AV(pt)+AL showed improvement when compared to the control and other treatment groups. This disagrees with the findings of Oyeyemi et al, [38] who reported a decrease in the motility of buck’s sperm cells treated with AV. Sperm cells livability were significantly increased in groups treated with AV alone and AV(pt)+AL compared to other groups. Our data showed that pre-treatment with AV conferred more protection to testicular tissues compared to concurrent treatment with AV in the male Wistar rat. Thus the higher sperm percentage motility and live/dead ratio in the former suggests a possible priming and buffering of the host antioxidant defense by AV, thereby mitigating redox dyshomeostasis [25]. AV possesses anti-apoptotic properties, thus increasing the livability of spermatogenic and androgenic cells in the testes of mice [28]. Percentage abnormalities were significantly lower in AV alone and AV(pt)+AL treated rats; an observation that contrasts with findings of Oyeyemi and Ajani [40]. They reported an increase in sperm abnormalities of albino rats (Wistar strain) following administration of aqueous extract of AV. These observed differences might be as a result of the different extraction methods adopted. Ethanol extract of AV, used in this study, has been shown to yield large amounts of flavonoids (antioxidants) [31]. The increase in percentage abnormalities noticed in animals treated with AL alone reveals AL-induced toxicity. This was, however, greatly improved in AV(pt)+AL groups; a phenomenon suggestive of the protective or ameliorative role of AV in AL-induced testicular toxicity. High percentage abnormalities score recorded in AV+AL(conc.) groups reflect a possibility that concurrent usage of AL and AV may reduce AV potency to confer protection on the testis.

The testicular histopathology recorded in testes of rats dosed with AL alone demonstrated as marked destruction of the germinal epithelium and mineralization of the luminal content of the seminiferous tubules (with no evidence of spermatogenesis) as well as granulomatous reactions in the interstitium is in consonance with findings of Adekunle et al, [9]. The destructive effect of AL on testicular tissue might have been as a result of the presence of the endoperoxide bridge responsible for production of free radicals [41]. Free radicals cause oxidative stress in the testes, lipid peroxidation and ultimately, to its inability to produce viable sperm cells [14,25-27]. This observation is further strengthened by the strong iNOS signal intensity (a marker for oxidative stress) recorded in the group. AV-dosed rats’ testicular morphology appeared normal with mild desquamation of the germinal epithelium but with evidence of spermatogenesis; a likely presence of prooxidants in extracts of AV [43]. This may account for the low levels of iNOS signal intensity observed in this group. Testicular cytoarchitecture in AL-dosed rat was greatly improved in AV+AL (conc.) treated rats owning to the antioxidative property of AV [20, 31, 44]. This was reflected in iNOS signal intensity of this group as it was very close to the control group. The role of AV in protecting testicular tissues against oxidative damages induced by toxic chemicals such as Bisphenol A [25], cisplatin [26] and irradiations (x-rays) [27] have been well documented. AV exerts its anti-oxidative properties by scavenging free radicals and buffering the cellular antioxidant defence machinery. Our data agrees with a study conducted by Behmanesh et al, [45], who noted that AV increased the antioxidant defence while mopping up free radicals in Wistar rats.

The mild disruption of the testicular cytoarchitecture and the lower levels of iNOS signalling in AV(pt)+AL group compared to AL group might be as a result of prior priming of the rodents’ defense mechanism against AL-mediated testicular toxicity. It is also not unlikely that the observed pathologies were due to the discontinuance of AV.

From this study, it appeared AV had a stronger affinity for the epididymis and its content compared to the testis. Thus, conferring better level of protection on the epididymis (spermiogram) compared to the testicular tissues (seminiferous tubules). The pathologies observed in testicular interstitium of AL-dosed rats did not significantly affect testosterone levels. However, the destruction of the testosterone-producing Leydig cells and granulomatous reaction seen in the interstitium portends a strong likelihood that it might affect testosterone levels following chronic use and/or abuse of this drug especially in younger subject.

## Conclusions

The concurrent administration of AV and AL protected testicular architecture while the use of AV prior AL administration improved the spermiogram of albino rats (Wistar strain). AL induces testicular pathology, thus should be used with care in male subjects. AV can confer a level of protection against these defects if used prior to administration of the drug.
